# Changes of retinal ganglion cell complex after vitrectomy in rhegmatogenous retinal detachment patients and its correlation with inflammatory blood biomarkers

**DOI:** 10.1186/s12886-022-02512-w

**Published:** 2022-07-02

**Authors:** Jiayi Song, Ting Chen, Wen Zuo, Wenyu Chen, Min Lei, Ming Ai

**Affiliations:** Department of Ophthalmology, Renmin Hospital of Wuhan University, No. 9 ZhangZhiDong Street, Wuchang District, Wuhan, 430060 Hubei China

**Keywords:** Rhegmatogenous retinal detachment, Vitrectomy, Ganglion cell complex, Inflammatory blood biomarker, Silicone oil

## Abstract

**Purpose:**

To compare retinal ganglion cell complex (GCC) parameters between rhegmatogenous retinal detachment (RRD) eyes and normal contralateral eyes after vitrectomy and to evaluate their correlation with inflammatory blood markers.

**Methods:**

We investigated 25 eyes that underwent vitrectomy due to RRD. Venous blood samples were collected from all participants before 08:00 a.m. on the second day of admission after a 12-hour fast for blood counts. The differences of retinal structure between RRD and contralateral eyes were compared 1 week postoperatively.

**Results:**

Focal loss volume (FLV) (2.009 ± 1.286)% was significantly increased compared with the contralateral eyes (*p* <  0.001). Monocyte-to-high-density lipoprotein was significantly positively correlated with GCC thickness parameters, and negatively correlated with FLV and global loss volume (GLV). Postoperative best-corrected visual acuity was negatively correlated with GLV (*p* = 0.039, *R*^2^ = 0.172).

**Conclusion:**

Retinal ganglion cells (RGCs) loss might present early postoperatively in RRD eyes, and was associated with systemic inflammation. RGCs loss might affect postoperative vision.

## Introduction

Rhegmatogenous retinal detachment (RRD) is a vision-threatening ocular disease caused by the separation of the neurosensory retina from the retinal pigment epithelium (RPE) due to retinal breaks [1]. Surgical treatment can anatomically reattach the detached retina, but postoperative vision recovery may not be satisfactory, especially in patients with macular involvement [2]. It has been reported that when RRD occurs, the hypoxic damage caused by the separation of the neurosensory retina from the RPE and the ischemic damage caused by the increase of inflammatory mediators combine to make postoperative visual loss [3, 4].

Inner layers of the retina are highly sensitive to hypoxia, especially retinal ganglion cells (RGCs) [5]. When RRD occurs, hypoxia impairs the normal physiological functions of the retina. Retinal hypoxia is associated with the RGCs loss, and is considered to be the basis of sight-threatening complications, which occurs in ophthalmic diseases such as glaucoma, ischemic optic neuropathy and diabetic retinopathy (DR) [4]. Toxicity and ischemia of silicone oil (SO), a filler used in retinal reattachment surgery, can also lead to damage of RGCs and internuclear synapses [6, 7]. After retinal reattachment, ganglion cell layer -inner plexiform layer complex (GCL-IPL) thickness is negatively correlated with the postoperative time, which may be related to the continuous RGCs loss [8]. Death of RGCs has been observed in animal model of RRD particularly in the detached retina [9, 10], but the loss of RGCs in human RRD patients has not been sufficiently studied. In addition, the level of inflammatory cytokines in the vitreous fluid increases after RRD [11]. And excessive production of inflammatory cytokines also accelerates RGCs death [4].

The axons, cell bodies and dendrites of RGCs are located in the retinal nerve fiber layer (RNFL), GCL and IPL respectively, and form the retinal ganglion cell complex (GCC). The GCC parameters can be measured non-invasively using spectral domain optical coherence tomography (SD-OCT). In terms of systemic inflammation, complete blood count (CBC) parameters such as platelet-to-lymphocyte ratio (PLR), neutrophil-to-lymphocyte ratio (NLR) and mean platelet volume (MPV)-to-platelet ratio (MPR) have been regarded as cheap and convenient markers recently [12]. Those markers have been investigated to predict the severity of ophthalmic diseases such as glaucoma, DR and retinal vein occlusion (RVO) [13–15].

Therefore, we compared the GCC parameters between the study eyes after pars plana vitrectomy (PPV) and the normal contralateral eyes in the early postoperative period, and evaluated their correlation with systemic inflammation markers. Furthermore, factors related to postoperative vision were also evaluated.

## Materials and methods

This cross-sectional observational study was approved by the Ethical Committee Board of Renmin Hospital of Wuhan University in accordance with the tenets of the Declaration of Helsinki. Informed consent was obtained prior to the examination. We examined patients who underwent PPV with SO tamponade for a primary unilateral RRD between January 2020 to May 2021 in Renmin Hospital of Wuhan University.

The inclusion criteria were patients with primary unilateral macular-off RRD within 60 days of onset who underwent PPV with SO tamponade. Criteria for the exclusion were as follows: (1) traumatic or tractional retinal detachment, (2) any previous or concomitant ocular diseases in either eye (e.g., glaucoma, uveitis, DR, macular degeneration), (3) previous intraocular surgery or retinal laser photocoagulation, (4) proliferative vitreoretinopathy (PVR) grade greater than C [16], (5) high myopia (axial length ≥ 28.0 mm) or the difference between the axial length of both eyes is greater than 1.0 mm, (6) retinal detachment remained 1 week postoperatively, (7) severe media opacities, the SS-OCT image quality score < 40 or measurement errors, (8) less than 18 years old. In addition, systemic diseases such as kidney disease, cardiovascular disease, tumors, chronic obstructive pulmonary disease, and uncontrolled hypertension were excluded.

All patients were questioned in detail for medical history, and underwent comprehensive ophthalmic examinations, including best-corrected visual acuity (BCVA), non-contact intraocular pressure measurement (IOP), slit lamp microscopy, indirect ophthalmoscopy, ocular B-scan ultrasonography, axial length, wide-field fundus photography (Optos P200Tx), and SD-OCT (RTVue XR AngioVue Version 2017.1; Optovue Inc. Fremont, CA, USA). For statistical analysis, BCVA was converted into the logarithm of the minimum angle of resolution (logMAR). Preoperative preparation also included collecting peripheral blood samples for laboratory testing before 08:00 a.m. on the second day of admission after a 12-hour fast. All ocular evaluations were repeated 1 week postoperatively and compared with the normal contralateral eye.

### Surgical procedure

A standard 3-port 23G PPV was performed in all patients under local anaesthesia. After vitreous removal, air-fluid exchange and endolaser photocoagulation around the retinal breaks were performed. Then, SO tamponade was performed. All operations were performed jointly by two experienced retinal surgeons (MA and TC). The SO was removed when a stable retinal attachment was observed 3 months after SO tamponade.

### Image acquisition

RTVue XR SD-OCT system was used to assess macular structure. Central foveal macular thickness (CFT) was assessed by “macular OCT mode”, which covered a 5 × 5 mm area centred at the fovea. The fovea was defined as the central 1.00 mm circle of Early Treatment Diabetic Retinopathy Study gird. And the parafoveal was defined as an annular area centered on the fovea with inner and outer ring diameters of 1.00 and 3.00 mm, respectively. The thickness of the macula for the superior and inferior eye hemisphere were also measured.

**Fig. 1:**
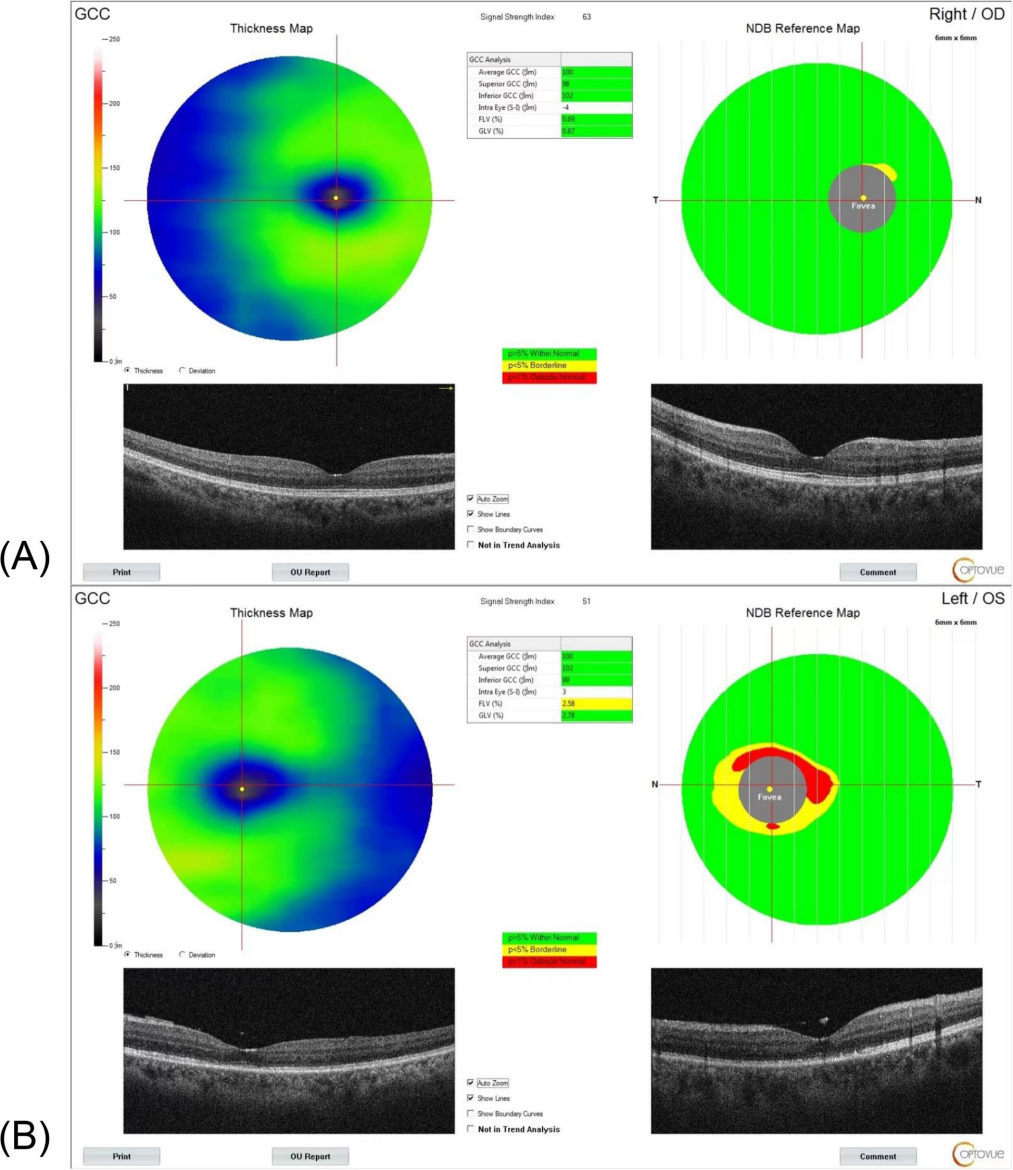
A representative case of a 51-year-old female patient with macula-off RRD in her left eye. **A** GCC parameters image of the contralateral eye, the avgGCC thickness was 100 μm, the supGCC thickness was 98 μm, the infGCC thickness was 102 μm, FLV was 0.69 and GLV was 0.87. **B** One week after the fovea was attached successfully, GCC parameters image of the RRD eye. The avgGCC thickness was 100 μm, the supGCC thickness was 102 μm, the infGCC thickness was 99 μm, FLV was 2.58 and GLV was 2.78

GCC thickness was performed by “Nerve Fiber-GCC mode”, scanning the distance between the internal limiting membrane (ILM) and the external boundary of the IPL. The GCC protocol consisted of a horizontal scan line of 7 mm and 15 vertical scan lines at 0.5 mm intervals within a 6 × 6 mm scan area centered 1 mm temporal to the fovea. The device automatically identified the GCC composed of the macular RNFL, GCL, and IPL, and obtained the following parameters: the average thickness of both sectors (avgGCC), the superior sector GCC thickness (supGCC), the inferior sector GCC thickness (infGCC), global loss volume (GLV) and focal loss volume (FLV) (Fig. 1).

### Statistical analysis

Shapiro-Wilk test was used to test the normal distribution of data. Quantitative data were described as mean ± SD, and compared between the RRD group and control group with t test or the Wilcoxon test. Spearman correlation analysis was used to evaluate the correlation between GCC parameters and laboratory parameters. The correlation between BCVA (logMAR) and clinical features, laboratory parameters, and SD-OCT parameters was then investigated using stepwise multivariate analysis. All statistical analyses were performed using IBM SPSS Statistics (version 25.0), and a *P*-value < 0.05 was considered statistically significant.

## Results

Fifty-three patients were initially enrolled in the study, of whom 17 patients had low image quality scores in study eyes, 3 patients had low scores in contralateral eyes, 3 patients had measurement errors, 3 patients had incomplete clinical data, 2 patients had retinal hole in contralateral eyes. Of the remaining 25 patients (12 males, 13 females), 25 study eyes were compared with the normal contralateral eyes. Clinical features and laboratory parameters were presented in Table 1. The mean age of RRD patients was 55.24 ± 10.77 years (range 31–75 years), and the average duration of RRD before surgery was 16.80 ± 15.40 days (range 2–60 days). All patients had postoperative retinal anatomical reattachment, and no obvious intraoperative or postoperative complications were observed, such as choroid hemorrhage, vitreous hemorrhage, endophthalmitis. None of the patients underwent cataract surgery before or during PPV. The mean postoperative BCVA (logMAR) was better than preoperative BCVA (logMAR) (*P* = 0.148) in study eyes. And there was no significant difference between preoperative IOP (15.48 ± 3.24 mmHg) and postoperative IOP (16.04 ± 3.92 mmHg, *P* = 0.542). IOP increased in 9 eyes (36.0%) within 1 week after surgery, all of which could be controlled by topical anti-glaucoma medication.

**Table 1:** Clinical features and laboratory parameters of the study participants

Variable	
Patients	25
Age (years)	55.24 ± 10.77
Gender (male/female)	12 / 13 (48.0% / 52.0%)
Diabetes (yes/no)	1 / 24 (4.0% / 96.0%)
Controllable hypertension (yes/no)	8 / 17 (32.0% / 68.0%)
Duration of RRD before surgery (days)	16.80 ± 15.40
Number of quadrants involved	2.13 ± 0.80
Preoperative BCVA (logMAR)	1.30 ± 0.78
Postoperative BCVA (logMAR)	1.00 ± 0.36
Preoperative IOP (mmHg)	15.48 ± 3.24
Postoperative IOP (mmHg)	16.04 ± 3.92
IIOP within a week	9 (36.0%)
Laboratory parameters	
White blood cell (× 10^9^/L)	5.67 ± 1.51
Neutrophil (× 10^9^/L)	3.46 ± 1.19
Lymphocyte (× 10^9^/L)	1.69 ± 0.60
Monocyte (× 10^9^/L)	0.40 ± 0.15
Platelet (× 10^9^/L)	222.76 ± 54.80
MPV (fL)	10.73 ± 1.02
TG (mmol/L)	1.28 ± 0.66
TCh (mmol/L)	4.60 ± 0.94
HDL-Ch (mmol/L)	1.39 ± 0.32
NLR	2.28 ± 1.24
PLR	141.99 ± 57.42
LMR	4.69 ± 2.09
MPR	0.05 ± 0.02
MHR (× 10^9^/mmol)	0.32 ± 0.17

*RRD* rhegmatogenous retinal detachment; *BCVA* best-corrected visual acuity; *logMAR* logarithm of minimal angle of resolution; *IOP* intraocular pressure; *IIOP* increased intraocular pressure; *MPV* mean platelet volume; *TG* triglyceride; *TCh* total cholesterol; *HDL-Ch* high density lipoprotein cholesterol; *NLR* neutrophil-to-lymphocyte ratio; *PLR* platelet-to-lymphocyte ratio; *LMR* lymphocyte-to-monocyte ratio; *MPR* MPV-to-platelet ratio; *MHR* monocyte-to-high-density lipoprotein

Comparisons of retinal thickness and GCC parameters between the study eyes 1 week postoperatively and the contralateral eyes are shown in Table 2. One week postoperatively, the mean thickness of the macular fovea, parafovea, superior and inferior hemisphere parafovea of the study eyes were significantly lower than that of the contralateral eyes (*p* = 0.026, *p* <  0.001, *p* = 0.001, *p* <  0.001, respectively). There were no significant differences in avgGCC (99.96 ± 10.86 μm vs. 98.60 ± 8.30 μm, *p* = 0.448), supGCC (100.56 ± 10.89 μm vs. 99.04 ± 7.86 μm, *p* = 0.294) or infGCC (99.60 ± 11.83 μm vs. 98.08 ± 9.31 μm, *p* = 0.726) between the study eyes and the contralateral eyes. FLV was significantly higher in the study eyes (2.009 ± 1.286)% than in the contralateral eyes (0.875 ± 1.065)% (*p* <  0.001). GLV was also higher in the study eyes (3.586 ± 3.346) % than in the contralateral eyes (2.624 ± 2.597) %, but not statistically significant (*p* = 0.052).

**Table 2:** Comparison of macular thickness and GCC parameters in study eyes 1 week postoperatively and normal contralateral eyes

	Study eyes	Fellow eyes	*P*-value
CFT			
Fovea (μm)	224.20 ± 46.26	248.52 ± 25.43	**0.026**
Parafoveal (μm)	289.84 ± 23.69	313.20 ± 17.64	**< 0.001**
supParafoveal (μm)	291.36 ± 26.86	313.96 ± 18.37	**0.001**
infParafoveal (μm)	288.52 ± 23.08	312.40 ± 17.51	**< 0.001**
GCC			
avgGCC (μm)	99.96 ± 10.86	98.60 ± 8.30	0.448
supGCC (μm)	100.56 ± 10.89	99.04 ± 7.86	0.294
infGCC (μm)	99.60 ± 11.83	98.08 ± 9.31	0.726
FLV (%)	2.009 ± 1.286	0.875 ± 1.065	**< 0.001**
GLV (%)	3.586 ± 3.346	2.624 ± 2.597	0.052

*GCC* Ganglion Cell Complex; *CFT* central foveal macular thickness; *FLV* focal loss volume; *GLV* global loss volume

*P* values in bold are significant

**Fig. 2:**
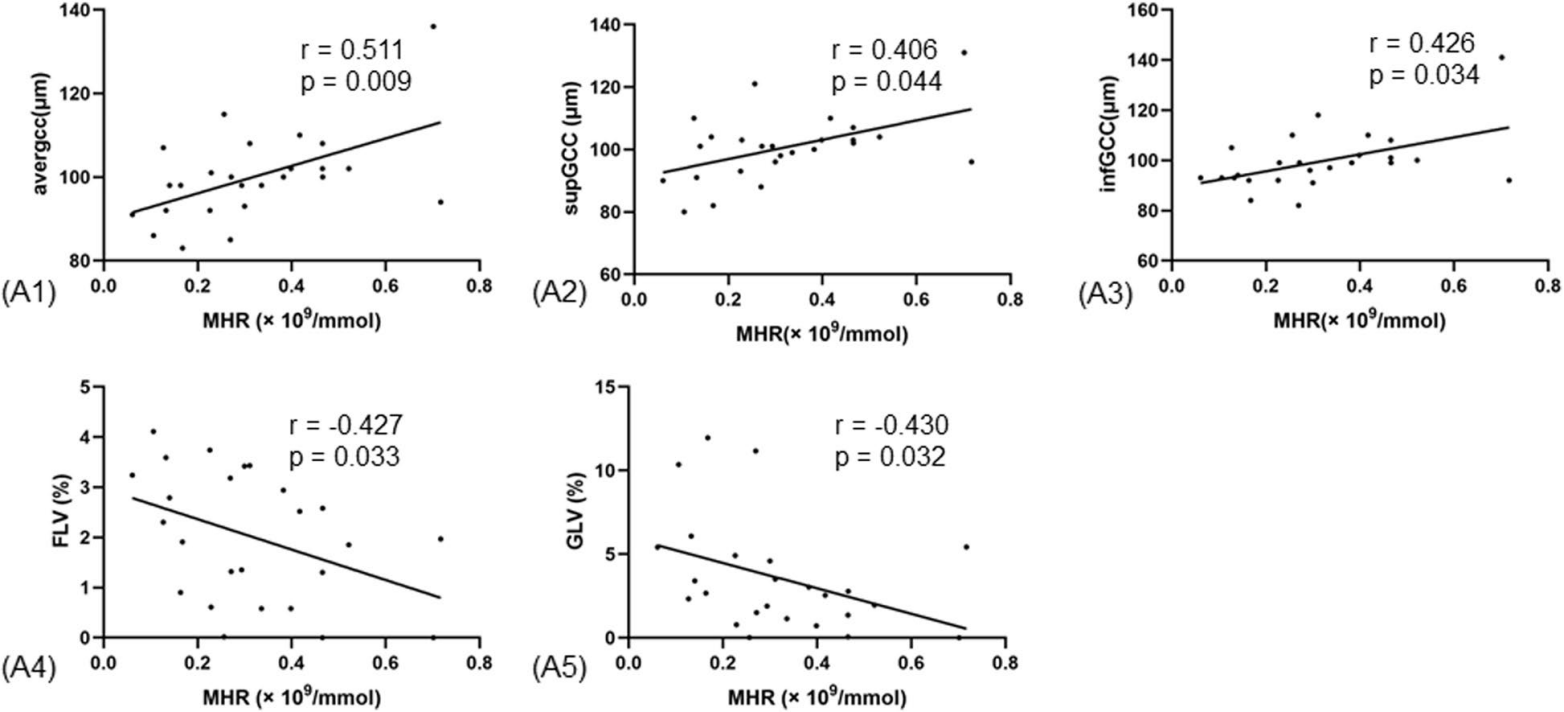
Correlation between MHR and GCC parameters in RRD patients. (A1, A2, A3, A4, A5) Correlation of MHR with GCC parameters including avgGCC thickness, supGCC thickness, infGCC thickness, FLV and GLV. Abbreviations: MHR, monocyte-to-high-density lipoprotein; GCC, ganglion cell complex; RRD, rhegmatic retinal detachment; avg., average thickness of both sectors; sup, superior sector; inf, inferior sector; FLV, focal loss volume; GLV, global loss volume

Spearman correlation analysis between GCC parameters in study eyes and laboratory parameters showed that monocyte-to-high-density lipoprotein (MHR) was significantly positively correlated with avgGCC thickness (*r* = 0.511, *p* = 0.009), supGCC thickness (*r* = 0.406, *p* = 0.044) and infGCC thickness (*r* = 0.426, *p* = 0.034), and negatively correlated with FLV (*r* = − 0.427, *p* = 0.033) and GLV (*r* = − 0.430, *p* = 0.032) (Fig. 2). In addition, FLV was negatively correlated with parafoveal thickness (*r* = − 0.446, *p* = 0.026), especially suparafoveal thickness (*r* = − 0.481, *p* = 0.015) (Table 3). However, there was no significant correlation between the macular thickness of the study eyes and laboratory parameters. Meanwhile, postoperative logMAR BCVA was positively correlated with avgGCC thickness (*r* = 0.423, *p* = 0.035), supGCC thickness (*r* = 0.441, *p* = 0.027) and infGCC thickness (*r* = 0.408, *p* = 0.043).

**Table 3:** Correlation analysis of GCC parameters with laboratory parameters and macular thickness in study eyes

	avgGCC (μm)		supGCC (μm)		infGCC (μm)		FLV (%)		GLV (%)	
	r	*P*-value	r	*P*-value	r	*P*-value	r	*P*-value	r	*P*-value
**laboratory parameters**										
Monocyte (× 10^9^/L)	0.460^*^	0.021	0.355	0.082	0.382	0.060	− 0.413^*^	0.040	− 0.409^*^	0.042
TG (mmol/L)	0.393	0.052	0.384	0.058	0.280	0.175	−0.390	0.054	−0.397^*^	0.049
Tch (mmol/L)	−0.157	0.453	−0.110	0.601	−0.178	0.394	0.187	0.371	0.135	0.521
HDL-Ch (mmol/L)	−0.386	0.057	−0.256	0.217	−0.363	0.074	0.358	0.079	0.371	0.068
MHR (× 10^9^/mmol)	0.511^**^	0.009	0.406^*^	0.044	0.426^*^	0.034	−0.427^*^	0.033	−0.430^*^	0.032
**macular thickness**										
Fovea (μm)	0.186	0.373	0.321	0.118	0.103	0.624	−0.344	0.092	−0.171	0.413
Parafoveal (μm)	0.242	0.243	0.356	0.080	0.240	0.247	−0.446^*^	0.026	−0.361	0.076
supParafoveal (μm)	0.285	0.167	0.425	0.034^*^	0.231	0.268	−0.481^*^	0.015	−0.388	0.055
infParafoveal (μm)	0.196	0.348	0.250	0.228	0.268	0.196	−0.376	0.064	−0.320	0.119

*GCC* Ganglion Cell Complex; *FLV* focal loss volume; *GLV* global loss volume; *TG* triglyceride; *TCh* total cholesterol; *HDL-Ch* high density lipoprotein cholesterol; *MHR* monocyte-to-high-density lipoprotein

* Statistically significant values (*P* < 0.05)

**Statistically significant values (*P* < 0.001)

The univariate regression analysis showed that postoperative logMAR BCVA was significantly correlated with supGCC thickness (*p* = 0.041, R^2^ = 0.170) and GLV (*P* = 0.039, *R*^2^ = 0.172) in study eyes. Then stepwise multivariate regression analysis was performed including age, IOP, duration of RRD, laboratory parameters and SD-OCT parameters. The final results showed that postoperative logMAR BCVA was negatively correlated with GLV in study eyes (standardized B = − 0.414, *p* = 0.039, *R*^2^ = 0.172).

## Discussion

In this study, we compared CFT and GCC parameters between contralateral eyes and study eyes 1 week after PPV by using SD-OCT and evaluated their correlation with systemic inflammatory markers in RRD patients. Our study showed that, 1 week postoperatively, the CFT of study eyes was significantly thinner than that of contralateral eyes, and FLV and GLV were higher than the contralateral eyes. GCC parameters were correlated with MHR. The prognosis of vision was correlated with GLV. To our knowledge, there is currently no study using the combination of GCC parameters and systemic inflammatory markers to investigate visual recovery after PPV in RRD patients.

Damage to RGCs, which are responsible for the integration and transmission of visual signals, is associated with visual impairment. In Nerve Fiber-GCC mode, FLV and GLV are pattern analyses of GCC thickness，and are more sensitive than GCC thickness [17]. FLV is determined by generating a modular graph of the thickness value of each pixel across the scanned GCC area, representing the percentage of significant GCC loss [18]. By measuring the average diffuse GCC loss of the entire scanned GCC area, GLV represents the percentage of GCC thickness reduction at each pixel location compared to the age-matched canonical database [18]. In the diagnosis and monitoring of glaucoma, GCC parameters have been found to be indicators for detecting disease course and predicting disease progression [19, 20]. FLV was significantly higher in DR patients than in control group [21] and was an independent predictor of diabetic peripheral neuropathy [18]. In addition, changes in GCC parameters have been found in patients with systemic diseases such as chronic kidney disease [22], Alzheimer’s disease [23] and primary pulmonary hypertension [24], which may provide useful evidence for early detection of retinal nerve damage. Therefore, GCC parameters are considered to be the most sensitive indicators of any adverse factors affecting the retina [25].

Ma et al. reviewed medical records of 7 RRD patients with unexpected visual loss after SO removal, and found that the GCC thickness of the affected eyes decreased, and the GLV and FLV were significantly higher [26]. As previously mentioned, SO-related toxicity and ischemia may lead to damage of RGCs and internuclear synapses during SO tamponage, and affect the measurement of GCC parameters with increasing tamponage time. In contrast, we evaluated the clinical data of RRD patients early postoperatively, that is, 1 week after SO tamponade. The changes of OCT parameters in patients may be more correlated with ischemic and hypoxia after RRD. The mean postoperative BCVA improved, and all patients’ visual occlusion were eliminated and their vision were clearer postoperatively. Ten patients had lower postoperative BCVA, which was considered to be related to changes in ocular refraction after SO tamponade [27, 28]. In our study, GCC thickness in study eyes did not change, but FLV and GLV increased compared with the fellow eyes, and FLV was statistically significant. It indicates that RGCs loss may have occurred before GCC thickness decreased in RRD patients.

Our study showed that the postoperative mean CFT in study eyes was significantly lower than that in contralateral eyes, which was consistent with the results of Roohipoor et al. [29]. At their subsequent 3-month follow-up, there was no significant change in the thickness of the fovea and parafovea. Wang et al. observed 14 patients with macular-off RRD who underwent intraocular gas tamponade and found no-significant change in the thickness of the parafovea compared with the fellow eyes at 2, 4, 8, and 12 weeks postoperatively [30]. Hong et al. reviewed patients with macular-off RRD who underwent intraocular gas tamponade 6 months postoperatively and found that the central retinal thickness was significantly thinner in the detached eyes, while the thickness of the parafovea did not change significantly [31]. The reason for the above-mentioned different results is considered to be related to the difference in fillers. Previous studies [32–34] on retinal thickness after SO tamponade showed that the inner retinal layer was thinned after SO tamponade, and RGCs and IPL contributed most to this thinning. And it may be associated with unexplained vision loss. Meanwhile, we found that FLV was negatively correlated with the mean thickness of macular parafovea, especially superior hemisphere, indicating RGCs loss in the macular area was correlated with retinal thinning. Therefore, we speculate that due to the high sensitivity of RGCs, RRD patients have already experienced RGCs loss in the early postoperative period. When RGCs loss is further aggravated to the extent that GCC thickness is reduced, unexplained vision loss may occur.

We further evaluated the correlation between GCC parameters and systemic inflammation markers and found that GCC thickness parameters were significantly positively correlated with MHR, while FLV and GLV were the opposite. Meanwhile, postoperative logMAR BCVA was correlated with GLV. This may indicate that the inflammatory mechanism is related to RGCs loss in RRD patients, and RGCs loss may affect postoperative vision recovery. Monocytes release pro-inflammatory and pro-oxidative cytokines, which play an important role in inflammation and oxidative stress [35, 36]. In contrast, HDL-Ch has anti-inflammatory and antioxidant effects by inhibiting cytokine expression and inhibiting monocytes activation and extravasation [35]. MHR is a new indicator of inflammation and oxidative stress. It has been recognized as a predictor and prognostic marker in patients with endothelial dysfunction, chronic kidney disease and cardiovascular disease [37]. Previous study has shown that inflammatory mediators such as interleukin-8 (IL-8) and vascular endothelial growth factor (VEGF) were significantly elevated in vitreous of RRD patients [38]. In our other study, monocyte counts and MHR were significantly lower in RRD patients compared to healthy controls. This suggests that although RRD is a localized ocular disease, RRD patients may have systemic inflammation activation. Taken together, we could hypothesize that when RRD occurs, local and systemic oxidative stress and increased inflammatory mediators may jointly cause the apoptosis of RGCs and affect postoperative vision.

Our study has several limitations. First, the sample size was relatively small, and more patients are needed to further confirm our results. Secondly, it was cross-sectional and lacked follow-up visit to explain the changes of GCC parameters after SO tamponade. Third, although we excluded patients with axial length difference greater than 1.0 mm between the eyes, the GCC thickness comparison may still be affected. Fourth, factors that may influence GCC parameters and inflammatory markers, such as controllable hypertension, were not completely excluded when we included the population.

In conclusion, our study showed that retinal structure was altered in eyes with macula-off RRD receiving SO tamponade compared to contralateral eyes. RGCs loss is present in study eyes and is associated with systemic inflammation. RGCs loss affects postoperative vision. Further studies are warranted.

## Data Availability

The datasets generated and/or analyzed during the current study are not publicly available due to privacy concerns but are available from the corresponding author on reasonable request.
